# Top ten tips to manage patients after acute kidney injury

**DOI:** 10.1093/ckj/sfag104

**Published:** 2026-03-25

**Authors:** Nicholas M Selby, Sophie De Seigneux, Turgay Saritas, Vincenzo Cantaluppi, Ana Belén Sanz, Stanislas Faguer, Jose Antonio Lopes, Marlies Ostermann, Joana Gameiro

**Affiliations:** Centre for Kidney Research and Innovation, Academic Unit for Translational Medical Sciences, University of Nottingham, Royal Derby Hospital Campus, Derby, UK; Service of Nephrology and Hypertension, University hospital of Geneva, Geneva, Switzerland; Department of Nephrology and Clinical Immunology, University Hospital RWTH Aachen, Aachen, Germany; Nephrology and Kidney Transplantation Unit, Department of Translational Medicine (DIMET), University of Piemonte Orientale (UPO), “Maggiore della Carità” University Hospital, Novara, Italy; Laboratory of Experimental Nephrology, Instituto de Investigación Sanitaria-Fundacion Jimenez Diaz (IIS-FJD), Universidad Autonoma de Madrid, RICORS2040, Madrid, Spain; Department of Nephrology and Organ Transplantation, Intensive Care Unit, University Hospital of Toulouse, INSERM U1297 (Institute of Metabolic and Cardiovascular Diseases), French Intensive Care Renal Network, Toulouse, France; Nephrology and Renal Transplantation Department, Unidade Local de Saúde Santa Maria, Lisbon, Portugal; Department of Critical Care & Nephrology, Guy’s & St Thomas’ Hospital, London, UK; Nephrology and Renal Transplantation Department, Unidade Local de Saúde Santa Maria, Lisbon, Portugal

**Keywords:** acute kidney injury, chronic kidney disease, chronic renal insufficiency, RAASi, SGLT2i

## Abstract

Despite high mortality, most patients who experience an episode of acute kidney injury (AKI) survive to hospital discharge. These patients are at increased risk for chronic kidney disease (CKD), recurrent AKI, cardiovascular events, hospital readmission, and premature mortality. AKI is causally linked to some of these longer-term outcomes, particularly CKD, but non-causal associations also serve to identify vulnerable patients at higher risk. In both scenarios, there is a role for improved post-discharge AKI care. However, current evidence for how to deliver this is incomplete, and there are knowledge gaps around which interventions are effective, which patients may benefit the most, and the health-economic impact of new care pathways. Current guidelines recommend monitoring kidney function within 3 months of AKI for all patients, which is challenging to implement and fails to account for varying individual patient needs.

In this review, we suggest ‘top ten tips’ that underpin a pragmatic framework for post-AKI care, based on the best available evidence. With a central role for patient engagement and education, these include early follow-up for patients with incomplete renal recovery, structured assessment of kidney function (including urinary albumin: creatinine ratio, and where appropriate, cystatin C), guideline-directed medication optimization (in particular renin-angiotensin-aldosterone-system inhibitors and sodium-glucose co-transporter 2 inhibitors), cardiovascular risk management and consideration of biopsychosocial aspects of recovery. These are supported by clear and actionable discharge communication, and coordination with other specialities and primary care.

## INTRODUCTION

**Figure 1:**
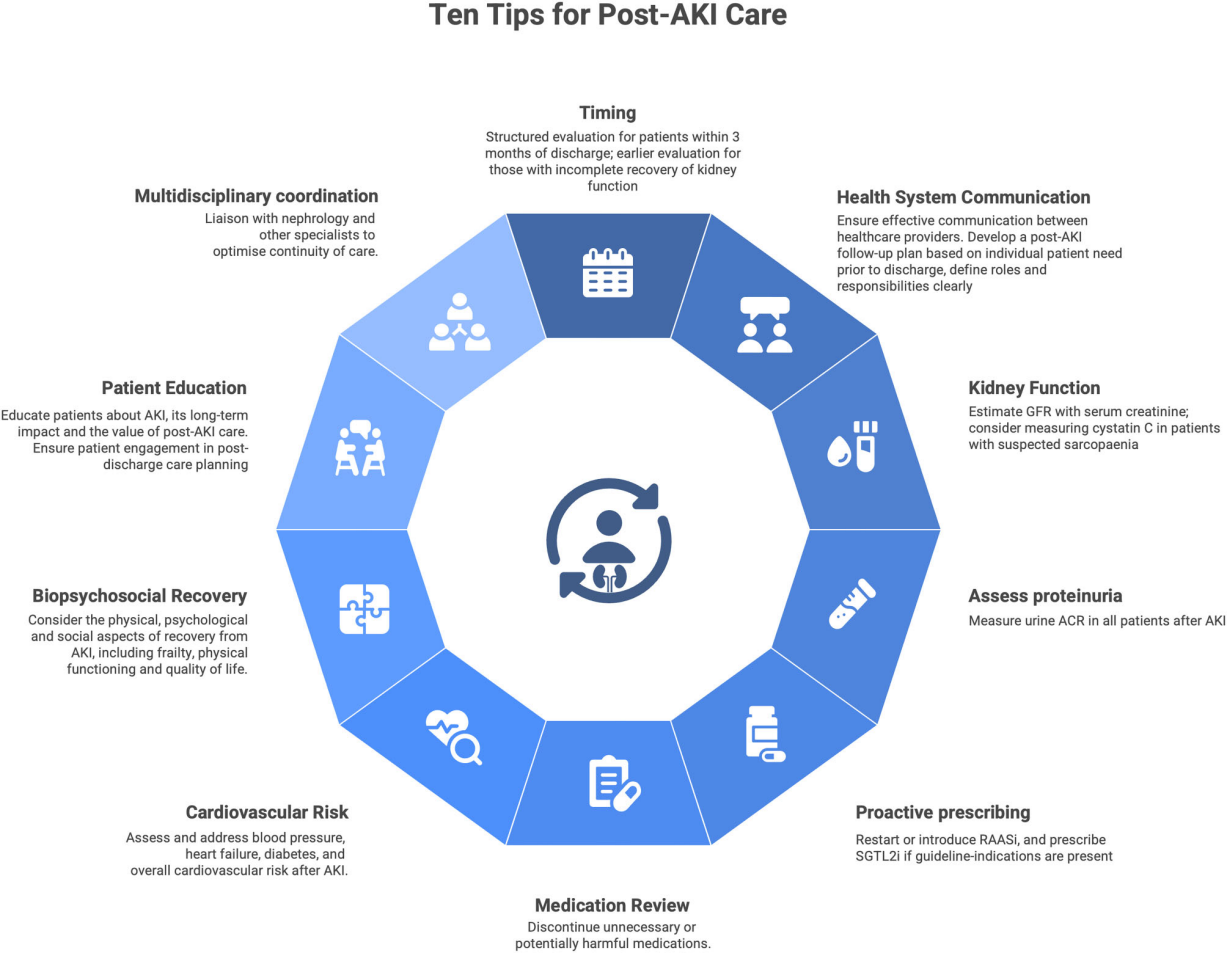
Summary of 10 ‘top tips’ for post-AKI care.

**Figure 2:**
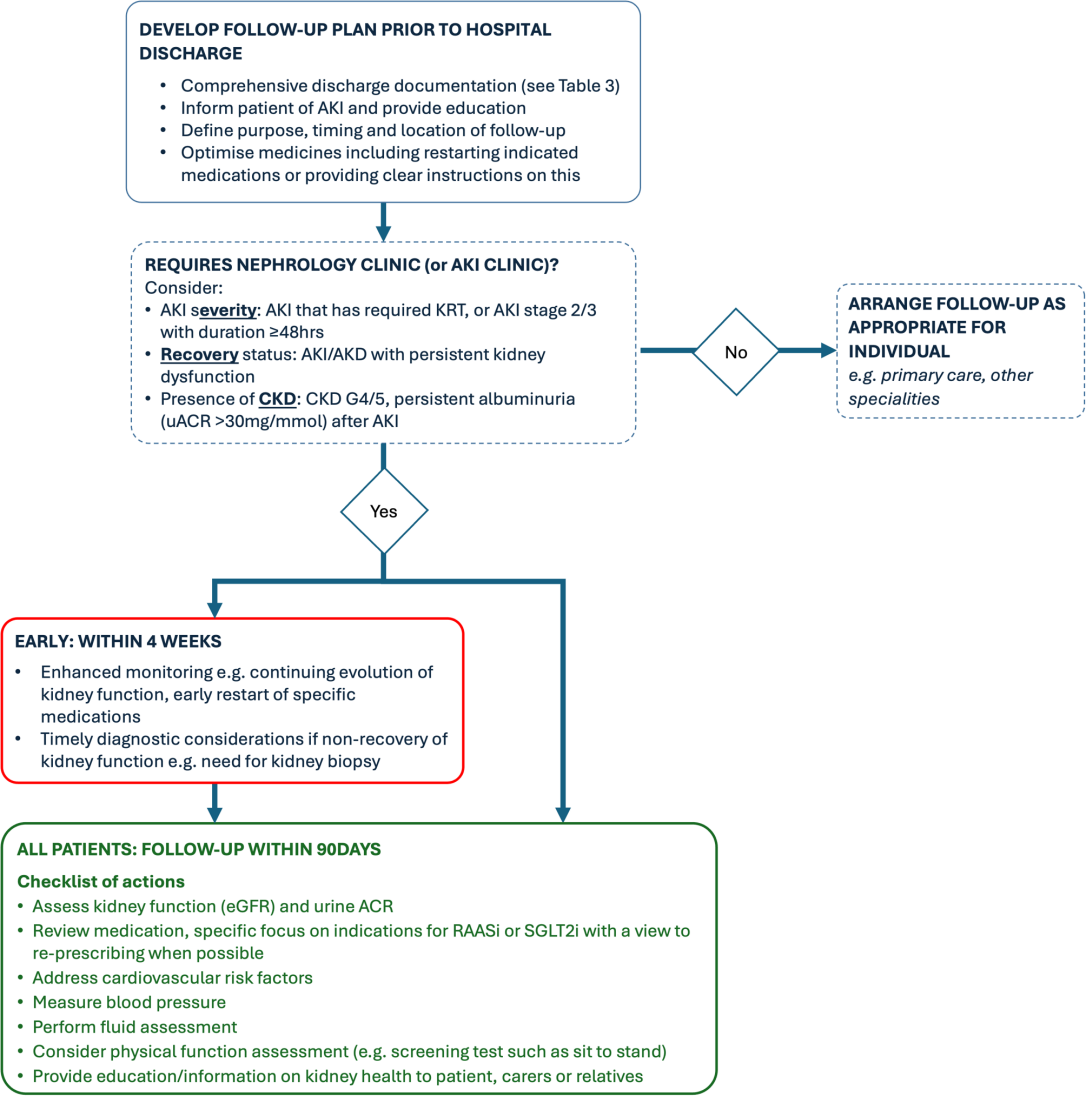
Flow diagram: a pragmatic approach to post-discharge AKI follow-up. (KRT = kidney replacement therapy; AKD = acute kidney disease; RAASi = renin-angiotensin-aldosterone-system inhibitors; SGLT2i = sodium-glucose co-transporter 2 inhibitors).

The high rates of short-term mortality seen in people with acute kidney injury (AKI) are widely appreciated. But even with mortality rates exceeding 20%, it follows that the majority of people who experience AKI survive to hospital discharge [1]. These people remain at substantial risk for a range of adverse outcomes, including development or progression of chronic kidney disease (CKD), recurrent AKI, hospital readmission, cardiovascular events, and premature mortality [2]; combined, these incur significant healthcare costs [3–5]. AKI is causally implicated in some of these longer-term health outcomes, particularly CKD, whereas in other scenarios an association with AKI identifies vulnerable people at higher risk [6]. In both cases, there is a logical argument that post-AKI care could be beneficial, and there are opportunities to make meaningful improvements as many patients receive little or no follow-up [7, 8]. However, there are challenges. The current evidence base is incomplete with few randomized controlled trials (RCTs) to inform which interventions improve outcomes after AKI, which patients will benefit from additional care, or the cost-effectiveness of new care pathways. Resultingly, current guidelines are hampered in making clear recommendations for patient management, with most limited to statements around measurement of kidney function within 3 months of the AKI episode (current guidelines are summarised in Table 1). These generally recommend that this should be done in all patients, and the CKD guidelines from the National Institute for Health and Care Excellence (NICE) in the UK go further, recommending ‘*monitoring of kidney function for 3 years after an episode of AKI*’ [9]. Such recommendations will likely exceed the capacity of many healthcare systems given the high incidence of AKI and the large numbers of patients that would require follow-up. Illustrating this, the UK Renal Registry reported 474 875 episodes of AKI associated with hospitalization in England in 2024, from which 325 078 people survived and would fall within the scope of current guidelines, generating multiples of this number in terms of appointments and laboratory tests [10]. Further, this approach fails conceptually to capture that individual patients have varying needs and may benefit from post-AKI care to different degrees. For example it remains unclear to what extent those with transient, stage 1 AKI need long-term follow-up. Here, we propose ‘ten tips’ as a practical guide for practitioners seeking to address this significant clinical need (that are summarised in Figs. 1 and 2), balanced against the lack of definitive evidence in some areas (level of evidence for each of the Top Ten Tips presented in Table 2).

**Table 1:** Summary of currently available (as of January 2026) guidance on post-AKI monitoring and follow-up, including guidelines from professional groups and toolkits/consensus statements.

Guideline group	Location	Year	Key post-AKI follow-up recommendations
*International and national guidelines*			
KDIGO AKI Guideline* [89]	International	2012	Reassess kidney function at 3 months to determine recovery vs CKD; intensity of follow-up depends on severity and recovery.
NICE AKI Guideline (NG148) [52]	United Kingdom	2024 update	Monitor serum creatinine after an episode of acute kidney injury. Base the frequency of monitoring on the stability and degree of renal function at the time of discharge. Consider referral to a nephrologist or paediatric nephrologist when eGFR is 30 ml/min/1.73 m^2^ or less in adults, children and young people who have recovered from AKI.
NICE AKI Quality Standard (QS76) [109]NICE AKI Quality Standard (QS76) [109]	United Kingdom	2023 update	Adults discharged from hospital after acute kidney injury have a clinical review within 3 months, or sooner if they are at higher risk of poor outcomes.
NICE CKD Guideline (NG203) [9]	United Kingdom	2021 update	Monitor adults, children and young people for the development or progression of CKD for at least 3 years after AKI (longer for people with AKI stage 3) even if eGFR has returned to baseline.
UK Kidney Association AKI Guidance UK Kidney Association AKI Guidance [110]	United Kingdom	2019	A discharge letter should be generated to include key information on the AKI episode and post-AKI care. Guidelines do not contain specific recommended actions for monitoring or interventions. Nephrology referral recommended for CKD stage G4 or higher.
*Toolkits and consensus statements*			
Think Kidneys AKI programme (legacy document) [111]	United Kingdom	2019	10 recommendations covering individualised care and timing of follow up, accurate discharge summaries, AKI coding in primary care, medication management, measuring eGFR and uACR, and incorporating quality improvement strategies.
Royal College of General Practitioners (RCGP) Royal College of General Practitioners (RCGP) [112]	United Kingdom	2025	Ensure coding of AKI in primary care record, optimize medications, measure eGFR and uACR 3-months after AKI and follow NICE CKD guidelines for those with CKD
ISN AKI Toolkit ISN AKI Toolkit [113]	International	Ongoing	Follow-up within 3 months to assess eGFR, albuminuria, urinalysis, blood pressure, medication review, and patient understanding of AKI. Some patients may need earlier follow up (suggested AKI stage 3, incomplete recovery, comorbid conditions). Some patients may need referral for specialist assessment.
ADQI Consensus Statements ADQI Consensus Statements [114]	International	2017	Timing and frequency of eGFR monitoring to be based on risk of future outcomes (higher risk defined as AKI persistence, comorbidity, recurrent AKI). Recommendations to inform patient of AKI, that AKI should be well documented, and optimisation of medications.
ASN Outpatient Care of Dialysis-Requiring AKI [115]	United States	2025	Specific advice for patients with ongoing kidney failure requiring dialysis after hospital discharge

Abbreviations: AKI, acute kidney injury; CKD, chronic kidney disease; eGFR, estimated glomerular filtration rate; NICE, National Institute for Health and Care Excellence; KDIGO, Kidney Disease: Improving Global Outcomes; uACR, urine albumin : creatinine ratio; ISN, International Society of Nephrology; ADQI, Acute Disease Quality Initiative; ASN, American Society of Nephrology.

*Of note the KDIGO AKI guidelines are due to be updated in 2026, scope of work available here.

**Table 2:** Summary table of the current level of evidence that supports each of the Top Ten Tips. These were graded according to the following approach: 1 = Strong recommendation, 2 = Weak/Conditional recommendation; and A = High certainty, B = Moderate certainty, C = Low certainty, D = Very low certainty. Three recommendations are classified as ‘Good practice statements’, which means that they do not fit into traditional approaches for grading evidence because there is no meaningful alternative, the recommendation is low risk, and will likely have high consensus.

	Recommendations	Certainty	Strength	Grade of evidence	Justification of evidence Grade
1	Provide clear discharge communication after AKI	Low	Strong	Good practice statement	System-level best practice; indirect evidence of benefit
2	Arrange early follow-up if incomplete recovery	Low	Conditional	2C	Observational assocations with incomplete recovery and adverse outcomes; confounding likely; no RCTs
3	Measure uACR after AKI	Moderate	Strong	1B	Strong prognostic value in AKI; indirect RCT evidence from non-AKI populations
4	Consider measuring cystatin C in suspected sarcopaenia	Low	Conditional	2C	Evidence for diagnostic superiority in sarcopaenia in non-AKI populations; limited evidence and no diagnostic accuracy studies in AKI
5	(Re)start RAASi if guideline-indication present	Moderate	Strong	1B	High-quality RCT base outside AKI; observational data only in post-AKI populations
6	Prescribe SGLT2i if indicated post-AKI	High–Moderate	Strong	1A–1B	Robust RCT data; no RCTs in post-AKI populations (particularly those without other indications)
7	Assess/address cardiovascular risk	Moderate	Strong	1B	Strong observational assocations, strong RCTs in non-AKI populations
8	Consider biopsychosocial elements of recovery	Low–Very Low	Conditional	2C–2D	Mainly qualitative and extrapolated evidence
9	Provide kidney health education	Low	Strong	Good practice statement	Minimal harm; strong consensus; unlikely to be RCT data
10	Liaise with specialist services	Low	Strong	Good practice statement	System-level optimisation; minimsl harm, strong consensus

### Provide clear and actionable discharge communication after AKI

The first step in successful transition of care after hospitalization is communication between hospital teams, primary care, and patients. Awareness of an AKI episode remains insufficient among clinicians and patients, often leading to missed opportunities for follow-up. In many patients, discharge summaries omit key information and lack instructions on post-discharge care, whereas clear documentation of an AKI episode is associated with fewer hospital readmissions [11–13]. This lack of communication is one factor contributing to the small proportion of patients who currently receive post-AKI assessments [14–16].

The discharge summary should document that an episode of AKI occurred, its severity and duration, presumed underlying cause, details about hospital course, requirement for dialysis, baseline kidney function, and the degree of kidney function recovery at discharge (these are summarised in Table 3) [17]. These details are important in determining follow-up intensity and support diagnostic coding of AKI in the primary care patient record to improve individual management. Additionally, the discharge summary should include specific and actionable instructions on timing and setting of follow-up, monitoring of kidney function, and prescribing information [17]. Few would disagree with this, but the challenge comes in implementation. Discharge summaries are commonly generated by junior staff under time and workload pressure, sometimes lacking clinical information or clear direction from senior clinicians [18]. System changes may therefore be required if robust follow-up plans are to be developed prior to discharge. Fundamentally, coordination of care involving both primary and secondary care services underpins the delivery of structured, value-adding assessments within three months of discharge for the majority of those with AKI, while enabling early, targeted follow-up for those who require it. Individualized follow-up arrangements with patient engagement and clear delegation of responsibilities, a risk-based follow-up schedule, and proactive medication management are also key elements. Further research including implementation science approaches are needed to design and test different care pathways that can function in different clinical contexts (e.g. shared care across primary and secondary settings, nephrology follow-up, nurse-led AKI clinics, multidisciplinary approaches, role of virtual consultations) [19–22].

**Table 3:** Example of information that should be included in comprehensive discharge documentation after an episode of AKI.

Statement that AKI has occurred during this hospital admission
Duration of AKI (days)
Severity of AKI (peak AKI stage, highest serum creatinine)
Severity of AKI (Kidney Replacement Therapy)
Baseline kidney function prior to AKI (serum creatinine, eGFR)
Kidney function at discharge (serum creatinine, eGFR)
Timing and location of follow-up (primary or secondary care, specify secondary care arrangements made prior to discharge e.g. clinic details)
Instructions of monitoring requirements for first post-discharge appointment (eGFR, urine ACR, any outstanding investigations pending from inpatient stay)
Prescribing information, particularly clear instructions if RAASi, SGLT2i, statins need to be restarted, saying when and by who
Confirmation that the patient has been informed of the AKI episode and the follow-up arrangements that have been put in place

### Arrange early follow-up for patients with incomplete renal recovery

There are several reasons that may necessitate follow-up in the first few days or weeks after AKI, but an important group are those with incomplete recovery of kidney function. For some of these patients, there may be ongoing diagnostic considerations, some require more frequent monitoring because kidney function is still in flux, and others require early re-initiation or modification of medications [23]. Recognizing incomplete recovery is also important as it strongly associates with adverse long-term outcomes [24–26]. In a cohort of >190 000 patients, incomplete recovery of kidney function was associated with a significantly higher risk of mortality and adverse renal outcomes compared with those who recovered to within 25% of baseline creatinine [27]. This is relatively common, with recent studies reporting the incidence of Acute Kidney Disease (AKD, defined as persistence of AKI for ≥7 days) as high as 33% [28, 29]. In a recent meta-analysis, AKD was associated with a more than four-fold higher risk of CKD, a six-fold increase in kidney failure, and a 39% increase in mortality [29]. AKD is also associated with higher rates of heart failure hospitalization, ischaemic heart disease, peripheral artery disease, reduced quality of life, and poorer functional recovery after ischaemic stroke [30–33].

These findings emphasize the need to identify patients with incomplete renal recovery after AKI, as they represent a high-risk group requiring structured and timely follow-up. Earlier intervention aims to interrupt the transition from AKI to CKD, manage cardiovascular risk and improve long-term outcomes, and there is emerging evidence that this is deliverable. James *et al*. developed a risk prediction tool for the development of CKD stage 4 within 1 year of AKI [34]. After validation, this tool (accessible here) was then used in a trial that randomized 155 participants with AKI stage 2/3 to a three-tier risk-based intervention vs usual care. Results showed the intervention group had an improved combined primary endpoint [proportion of participants receiving a renin-angiotensin-aldosterone-system inhibitor (RAASi), statin, or nephrologist follow-up within 90-days] [20]. The improvement in prescribing was most clearly seen in the high-risk group who had been referred to nephrology. Separately, multidisciplinary post-discharge care provided within 4 weeks of discharge resulted in lower urine albumin-creatinine ratio (uACR) and better blood pressure control at 1 year, although the small sample size of this trial limits generalizability [35]. Further research is required to determine if these changes in processes of care and surrogate markers will translate into meaningful improvements in harder, patient-centred outcomes.

### Measure urinary albumin-creatinine ratio after AKI

Assessment of proteinuria is astonishingly underutilized to assess kidney health after AKI. Proteinuria increases after an episode of AKI, reflecting persistent damage that portends a subsequent decline in kidney function, although the degree to which this has been reported is variable [36, 37]. In a large, multicentre cohort, 33.9% of those without proteinuria at baseline developed proteinuria after hospital-acquired AKI, while levels increased in 17.4% of those with pre-existing proteinuria [38]. In a retrospective cohort of >9600 patients, the incidence of new onset proteinuria after AKI was as high as 62% [39], and in a retrospective case-control study, AKI was associated with a higher risk of developing proteinuria in the first 12-months after discharge as compared to a propensity-matched non-AKI cohort (OR 1.35–1.65) [36]. Moreover, the risk of developing proteinuria after AKI was higher with AKI severity or pre-existing CKD and was lower in patients treated with RAASi [36, 38, 39]. However, a combined analysis of CRIC and ASSESS-AKI studies concluded that an episode of AKI was associated with a more modest 9% increase in urine protein-to-creatinine ratio [37].

Crucially, quantification of proteinuria provides prognostic information on long-term outcomes. After AKI, proteinuria is associated with lower rates of recovery and increased risk of kidney disease progression [38]. In the prospective ASSESS-AKI study, higher uACR at 3 months was independently associated with an increased risk of kidney function decline, and was a stronger predictor than the estimated glomerular filtration rate (eGFR) [40]. Despite this, it is surprising how rarely uACR is monitored after AKI. In a retrospective cohort, only 12% had a quantitative measurement of proteinuria in the 12 months after hospitalization [16]. Brar *et al*. reported that uACR was measured in the first 90 days after AKI in <10% of people with pre-existing CKD and <5% in those with normal baseline kidney function [7]. Therefore, due to its prognostic value, widespread availability, low cost and importance in prescribing decisions, quantification of proteinuria using uACR should be an essential element of post-AKI care, with significant scope for wider adoption.

### Consider measuring cystatin C in patients with suspected sarcopaenia

Routine assessment of kidney function is typically based on the measurement of serum creatinine and blood urea nitrogen. In critically ill patients, however, loss of muscle mass has significant effects on serum creatinine independently of kidney function. This can result in overestimation of kidney function at hospital discharge [41, 42]. Critically ill patients lose, on average, 2% of skeletal muscle per day during the first week of admission [41]. In a retrospective analysis of critically ill patients, duration of hospitalization was independently associated with a 30% decrease in serum creatinine from baseline [43].

Cystatin C, which is not affected by muscle mass, may provide a more reliable assessment of kidney function [44]. In a retrospective study of critically ill patients, cystatin C eGFR (eGFR_CYS_) at discharge was significantly lower than eGFR_CRE_ (68 vs 92 ml/min/1.73 m^2^, *P* < .001) [45], while cystatin C, but not serum creatinine, was associated with increased 1-year mortality (HR 1.78, 1.46–2.18) [45]. The impact of muscle mass loss during hospitalization on GFR was also evaluated in a prospective study which demonstrated that each day in the intensive care unit (ICU) resulted in a predicted 2% decrease in muscle mass, and longer lengths of stay were associated with greater differences between eGFR_CRE_ and eGFR_CYS_ at a rate of 2 ml/min/1.73 m^2^/day [44].

Outside ICU populations, loss of muscle mass also occurs with discrepancies between eGFR_CRE_ and eGFR_CYS_ persisting for months after discharge. This raises additional questions about the accuracy of kidney function estimation during the early post-recovery period [46, 47]. The prospective AKI Risk in Derby study (in which only 6% of AKI patients had an ICU admission) reported that eGFR_CYS_ at 3-months after AKI was significantly lower than eGFR_CRE_ (53.4 vs 68.4 ml/min/1.73 m^2^), and this difference was more pronounced in the AKI group as compared to matched non-AKI group [48]. Results also showed that the difference between eGFR_CRE_ and eGFR_CYS_ has also prognostic utility [48]. Similarly, in ASSESS-AKI, eGFR_CYS_ ≤70% of eGFR_CRE_ was associated with higher risks of heart failure hospitalization, kidney failure, and mortality [49].

Although unaffected by muscle mass, cystatin C has its own non-GFR determinants including inflammation, thyroid disease, steroid use, and cancer, so both eGFR_CRE_ and eGFR_CYS_ could be inaccurate in the post-AKI period [44]. There are no studies that compare eGFR_CRE_ and eGFR_CYS_ to measured GFR or assessments of muscle mass in this setting. This means that while the discrepancy between the two is clearly documented, it is not currently known which is more accurate, how the combined cystatin-creatinine eGFR equation performs (most accurate in ambulatory populations), or how observed differences relate to underlying biological determinants. For example, higher cystatin C levels with inflammation, commonly associated with AKI and its recovery, could result in overly low estimations of GFR [50]. Whilst we await future research to address these important knowledge gaps, the Kidney Disease: Improving Global Outcomes (KDIGO) 2024 CKD guideline recommends ‘*using eGFR_CRE-CYS_ in clinical situations when eGFR_CRE_ is less accurate and GFR affects clinical decision-making,’* and this applies to selected patients recovering from AKI in whom there is clinical suspicion of significant muscle loss or evidence of sarcopaenia [51].

### (Re)start guideline-indicated medications, especially RAASi, at hospital discharge or at follow-up

RAASi are one of the categories of medications that are commonly stopped in patients at the time of AKI [13]. Often, this reflects a concern that RAASi may contribute to a reduction in GFR in the setting of hypovolaemia, hypotension or sepsis, or to manage hyperkalaemia that arises as a complication of AKI. A temporary cessation of these medications in these situations is recommended in guidelines [52], despite the evidence for this approach being inconclusive [53, 54]. Potential unintended consequences may arise if these medications are permanently stopped with loss of their established benefits in heart failure, diabetes or CKD. This is not uncommon—in a large cohort of more than 90 000 US veterans with AKI stage 2/3, RAASi were stopped and not restarted in almost a third of patients who were taking these at time of AKI [55], with similar findings reported elsewhere [56, 57]. There may be multiple reasons that contribute to this, including a fear of precipitating recurrent AKI linked to varying clinician confidence, as well as organizational factors relating to imperfect communication during the transition of care from hospital to the community [21].

Although there are no RCTs, consistent findings from observational studies support early reintroduction of RAASi following AKI [58]. Several large cohort studies have reported higher mortality and cardiovascular events in those who discontinue RAASi following AKI compared to those who continue, without differences in recurrent AKI [55, 59, 60]. Further, the timing of reintroduction may be important. Chen *et al*. compared outcomes in 5392 AKI survivors who restarted RAASi either at discharge, within 3 months, or at 4–6 months, with the latter group displaying higher rates of cardiovascular events, mortality and kidney failure [61]. Similar findings were seen in a cohort of US veterans with diabetes and proteinuria, where earlier reintroduction of RAASi after AKI was associated with lower mortality [62].

Results from observational studies should be interpreted with the knowledge that findings may be affected by residual confounding, and in some cases (e.g. those prone to recurrent volume depletion) the risk-benefit ratio for RAASi may favour a different course. However, for the majority of patients who have a guideline-recommended indication for their use, RAASi should be strongly considered after an episode of AKI, ideally at hospital discharge and if not, within the first 3 months, acknowledging the lack of randomized trial data in this setting. Reintroduction can be considered when the patient is not at risk of volume depletion or hypotension, serum potassium is ≤5 mmol/l, and with standard checking of kidney function 1–2 weeks later.

### Prescribe SGLT2i if a guideline-indication is present after AKI

Sodium-glucose cotransporter 2 inhibitors (SGLT2i) have been convincingly shown to lower the risk of AKI. Evidence comes from large placebo-controlled trials in populations with CKD, diabetes, and heart failure that include substantial numbers of non-diabetic patients, but not specifically post-AKI cohorts [63–66]. Meta-analyses of these RCTs report a reduction in AKI with a pooled hazard ratio of 0.77 (95% CI, 0.69–0.87), with benefit in both diabetic and non-diabetic populations [63–66]. In acutely unwell, hospitalized cohorts (including groups with heart failure), SGLT2i are associated with less AKI, reduced hospital readmissions and lower mortality, without an increase in adverse events [67]. Reduced AKI risk has also been reported after cardiac surgery [68], and during acute cardiac decompensation [69].

There are fewer studies in those who have had AKI, although extensive pre-clinical data have delineated mechanisms by which SGTL2i improve recovery after AKI, including preservation of mitochondrial integrity, regulation of metabolism and inflammation, and reduced tubular cell senescence [70]. A retrospective analysis of US veterans with diabetes and hospitalized with AKI showed that subsequent SGLT2i use was associated with lower mortality [71], reduced CKD progression and less AKI [72]. Recently, a double-blind, placebo-controlled trial of empagliflozin started 2–4 weeks after AKI stage 2/3 did not show a difference in the primary composite outcome of persistent kidney dysfunction, death or dialysis at 1 year, but did observe a reduction in the rate of recurrent AKI [73]. However, this trial was likely underpowered and follow-up too short to detect all important treatment effects. In addition to direct evidence, comorbidities such as CKD and diabetes are over-represented in those with AKI. Screening post-AKI populations for guideline-recommended SGLT2i indications will allow detection of treatment gaps and unveil opportunities for greater prescribing; considering CKD definitions, it makes most sense that this is done at 3 months after hospital discharge, accepting that in others (e.g. those with heart failure with reduced ejection fraction) the indication will be obvious earlier. Further trials in post-AKI cohorts are urgently required to determine if and when SGTL2i should be initiated across a broader range of people following AKI.

### Assess and address cardiovascular risk factors after an episode of AKI

Meta-analyses have reported 45%–48% increases in the relative risk of heart failure, 40%–45% increases in acute coronary syndrome/myocardial infarction (MI), and a 48% increase in major cardiovascular events (MACE) following AKI [74, 75]. AKI is associated with the development of elevated blood pressure in previously normotensive adults [76], although it is not conclusively proven that this is associated with cardiovascular outcomes [26, 77]. However, much of these data come from analyses of routine healthcare databases, and relative risk tells only part of the story. The prospective ASSESS-AKI study reported relatively low event rates of MACE over 5 years following AKI (1.5 per 100 patient years) that did not differ from the matched comparator group, but observed heart failure events more frequently [26]. In contrast, the rates of MACE within 1-year of an AKI episode in the CKD-REIN cohort were 8.1%, almost six-fold higher than the CKD comparator group without AKI [78]. The timing of different cardiovascular events may also vary, importantly with significant rates of hospital readmission due to heart failure in the first 30-days after AKI [79, 80]. Taken together, current observational data suggests that patients after AKI are at increased risk of cardiovascular events (either from pre-existing risk, comorbidities or potential consequences of AKI), but the nature and timing of specific cardiovascular outcomes is more variable (e.g. some studies report MACE at lower event rates as compared to CKD progression or mortality [6, 25, 26]).

Although there are no RCTs to determine whether interventions targeting cardiovascular risk specifically after AKI improve outcomes, many of these patients will sit in categories where screening for cardiovascular risk is already recommended based on age, established cardiovascular disease, other comorbidities (e.g. diabetes, CKD) or risk factors (smoking, obesity). There is also evidence that the prescribing of medications to reduce cardiovascular risk is lower than expected after AKI. In a mixed-methods study of post-AKI follow-up practices, 67% of nephrologists and 73% of primary care physicians surveyed did not recommend statin therapy in a scenario when a guideline-indication existed [19]. In a propensity-score matched study of patients with a history of MI, patients with subsequent AKI were significantly less likely to be prescribed a statin, beta-blocker or RAASi as compared to those without AKI [57].

Therefore, assessment of cardiovascular risk factors following AKI is justified based on an increased likelihood of adverse outcomes, high prevalence of cardiovascular risk factors, and lower than expected rates of prescribing. Risk assessment should comprise of a review of guideline-indicated medications, assessment of volume status in patients with heart failure, and lifestyle recommendations such as smoking cessation.

### Consider biopsychosocial elements of recovery from AKI

The recovery process from a prolonged hospital admission and serious illness is complex. Patients can face broader issues including reduced physical functioning, feelings of anxiety and vulnerability, financial uncertainties or social issues such as impaired life participation or additional strain in relationships [81]. Issues can be compounded if patients leave hospital unaware that they have had AKI, if they are uncertain about the diagnosis or what follow-up is required, or if there is confusion about how to manage their recovery [18]. These issues may also serve to down-prioritize a focus on kidney health and longer-term risk modification, particularly if there are ongoing, competing care needs from other chronic conditions.

Although these aspects are increasingly documented [81, 82], at present there is no robust evidence to inform if interventions across these domains are effective following AKI. Research is clearly needed, because it does not necessarily follow that the most obvious approaches will be of benefit. For example, in ICU survivors, benefits of enhanced physical rehabilitation on quality of life or mortality have not clearly been shown [83]. However, there are some simple things that do not need testing in RCTs, such as better information for patients and their relatives, and greater clarity and communication about the organization and aims of follow-up. Sometimes, simply acknowledging and discussing these issues can help. Other patients may benefit from psychology or social work support.

### Provide education to all patients, ‘why kidney health matters’

Some patients with severe AKI have undergone a prolonged stay in the ICU or intermediate care, often characterized as a ‘story with gaps,’ where patients recall a mix of real and unreal events, intense emotions, and sometimes complete memory loss. The nature and quality of these recollections are influenced by factors such as delirium, depth of sedation, length of stay, and individual patient characteristics [84–87]. Outside of ICU, AKI is often poorly understood and incompletely remembered by patients as the concept is complex, relies on laboratory values, and can be perceived as a transient event. Even among patients with severe AKI (stage 2 or 3), understanding of the diagnosis, its causes, and implications is highly variable and frequently minimal [88]. As such, the KDIGO AKI guideline highlights the importance of patient education for individuals recovering from AKI [89], and this is a vital element to improve patient engagement in post-AKI care plans.

‘Sick day rules’ are commonly part of patient education, although at present, there is no robust evidence that this practice reduces subsequent AKI [90, 91]. These rules involve temporarily discontinuing medications that may precipitate or worsen AKI, such as RAASi, diuretics, or NSAIDs, during episodes of acute illness (e.g. vomiting, diarrhoea, fever, or dehydration) aiming to prevent recurrent AKI. Only one RCT has evaluated sick day protocols, finding no significant improvements in AKI incidence, change in kidney function or hospitalization rates [92, 93]. Consequently, there is currently insufficient high-quality evidence to support routine implementation of sick day rules after AKI. Whilst they may be appropriate for selected patients who are able to reliably enact them, this should be balanced with the risk that some patients may not restart their kidney and cardioprotective treatments.

### Liaise with other specialist services to optimize follow-up pathways

Expert consensus (Acute Disease Quality Initiative) recommends a multi-disciplinary approach to post-AKI care, which sometimes necessitates involvement of nephrology and non-nephrology specialists, as well as primary care [94]. However, there is often little coordination between nephrology and other specialist follow-up, as highlighted in a detailed analysis of patients’ lived experiences after AKI. Patients commonly received parallel follow-up appointments for post-AKI care and for other chronic conditions, reported that navigating and managing multiple follow-up appointments could be ‘a struggle,’ and that the number of appointments made it feel like they did not ‘really have the time or ability to be doing anything else’ [18]. This can be compounded if these follow-up appointments are poorly co-ordinated so that patients feel ‘feel lost and adrift’ in their aftercare or lack clarity on how AKI related aspects of care should be integrated into ongoing management [18]. Further, healthcare providers often perceive AKI as a complex condition to manage [21].

Pragmatically, it is important to recognize that within the whole AKI population, people will have different co-morbidities, challenges and priorities that influence their post-discharge care arrangements, further influenced by social circumstances and overall prognosis. For some groups with ongoing follow-up in non-nephrology specialities, it may be better to provide post-AKI care within this, improving clarity for patients, reducing healthcare burden by avoiding additional AKI/nephrology appointments and better integration of kidney health in their overall care. Obvious examples are heart failure and oncology, particularly if multidisciplinary outpatient services are already in existence [95–97]. For this to be successful, there are some prerequisites, including buy-in from the teams involved, capacity in the system to include kidney health within consultations, education of non-nephrology teams, guidelines to highlight what this should involve, and co-ordinated pre-discharge planning. In other scenarios, a focus on end-of-life care and co-ordination with palliative care may be needed. Underpinning this is the fundamental role of primary care in co-ordination of care.

### Future directions in risk stratification after AKI

This review focuses on practical recommendations, but post-AKI care is evolving. Definitions of renal recovery based on serum creatinine are flawed (discussed above), and furthermore do not capture changes in kidney functional reserve. After AKI, compensatory adaptations of glomerular hypertrophy and increased single-nephron GFR may maintain near-normal total GFR despite nephron loss, something not captured with current diagnostic tools. Reduced functional reserve may predispose to future insults, increasing the risk of AKI after cardiac surgery [98], and postulated to contribute to CKD [99]. This may be particularly relevant in paediatric AKI, where consequences may only manifest many years later. However, assessment of kidney functional reserve is currently cumbersome, so approaches better adapted for clinical settings would allow its wider study. Other opportunities may arise from biomarkers or imaging techniques that could bring additional information on underyling pathophysiology of the recovery process to improve risk stratification and aid decision-making. Biomarkers of tubular injury such as NGAL and urinary TIMP2*IGFBP7 when measured at time of AKI provide some information on likelihood of early recovery and survival up to 9-months [100, 101], with a range of biomarkers showing utility for prediction of subsequent CKD when measured immediately after cardiac surgery [102, 103]. Of more relevance, biomarkers have been identified during the recovery period after AKI that inform risk of long-term outcomes. In particular, soluble tumour necrosis factor receptor 1 and 2 have been identified in large, independent studies as having utility for prediction of subsequent CKD when measured 90-days after AKI [104–106], with recent external validation of these findings [107]. Clinical value seems most likely to be realized if these biomarker panels are used as a ‘rule out’ test to identify patients at low risk of long-term outcomes who can be spared unnecessary follow-up, a strategy that would help manage the very large numbers of people who survive an AKI episode and allow post-AKI management to be focussed on those most likely to benefit. This now needs to be tested in clinical trials. Renal MRI may also provide new insights into the pathophysiology of AKI by assessing kidney blood flow, perfusion, oxygenation, and changes in tissue microstructure. This offers new possibilities to describe the nature and severity of AKI, track the time-course to recovery or progression to CKD, and may ultimately provide a method to noninvasively assess response to new therapies [108].

## CONCLUSIONS

Undoubtedly, patients who survive AKI are at increased risk for a range of adverse long-term outcomes. Current care gaps are significant with a complex range of causes, some of which may require system change, resources, and improved co-ordination of care across services to better deliver patient-centred post-AKI care based on individualized need. While additional, ideally RCT evidence is needed, there are a number of proactive steps that can be taken now to better assess risk, provide monitoring, improve post-AKI prescribing and support more holistic aspects of patient recovery within treatment plans that incorporate patient wishes, likelihood of potential benefit and existing care provision.

## Data Availability

No new data were generated or analysed in support of this manuscript.
